# Momentary social interactions and affect in later life varied across the early stages of the COVID-19 pandemic

**DOI:** 10.1371/journal.pone.0267790

**Published:** 2022-04-29

**Authors:** Hio Wa Mak, Diana Wang, Arthur A. Stone

**Affiliations:** 1 Dornsife Center for Self-Report Science, Center for Economic and Social Research, University of Southern California, Los Angeles, California, United States of America; 2 Department of Psychology, University of Southern California, Los Angeles, California, United States of America; Public Library of Science, UNITED KINGDOM

## Abstract

The COVID-19 pandemic has impacted many different facets of life. The infectious nature of the disease has led to significant changes in social interactions in everyday life. The present study examined how older adults’ patterns of everyday momentary social interactions (i.e., with no one, partner, family, and friends) and their affect varied across the early stages of the pandemic and whether the magnitude of affective benefits associated with social interactions changed across time. A total of 188 adults aged 50 or above (M_*age*_ = 62.05) completed momentary assessments in early March, late March, May, and July 2020. Overall, older adults spent more time in solitude and less time interacting with their friends after the declaration of the pandemic. Further, negative affect (NA) spiked after the pandemic declaration and then returned to pre-pandemic level. Finally, momentary interactions with close social ties were consistently associated with higher positive affect (PA) and lower NA whereas momentary solitude was associated with lower PA, but not related to NA. The magnitude of associations between specific social interactions (or solitude) and affect varied across time, and the onset of the pandemic appeared associated with this variation. During the presumably most stressful period, solitude was not associated with lower PA and family interaction was not associated with higher PA as they were at other times. Further, interactions with friends seemed to have diminished affective benefits following the onset of the pandemic.

## Introduction

The COVID-19 pandemic and the resulting responses to it have significantly impacted various facets of life, including lower level of physical activity, higher levels of financial worry, health anxiety and loneliness, and poorer mental health [1–3]. One of the most obvious impacts of the pandemic is on the social aspects of daily life. The highly infectious nature of the disease has brought about large-scale interventions targeting everyday social interactions to slow its spread, including many state-wide stay-at-home orders, social distancing involving keeping at least six feet apart from anyone outside of one’s household, closing of restaurants and shops, working from home, and banning of gatherings with people outside of one’s household. Given that social interactions are fundamental to one’s well-being [4,5], measures aimed at slowing the spread of the disease should have a significant impact on individuals’ social behaviors and their emotional well-being. In fact, evidence suggests that stay-at-home and other social distancing measures are negatively affecting individuals’ mental health, including higher levels of depressive and anxiety symptoms, insomnia, and stress [1,6]. As a result of these measures, it is likely that individuals were interacting less with other people due to stay-at-home order or had substituted in-person social interactions with digital interactions [7]. Nevertheless, it is unclear how the onset of the pandemic was linked to changes in the patterns of interactions with various close social ties, levels of affect, and the affective benefits associated with social interactions in everyday life. This pandemic is, then, analogous to an enormous natural experiment on social interaction that has brought about unique opportunities to understand social relationships and affective well-being.

### Social connection and well-being

Humans have a fundamental need for social connection, especially in close relationships [4]. The social convoy model postulates that social relationships are shaped by personal and situational factors that change over the lifetime and influence well-being through the structure and quality of the ties [8]. One structural change that occurs over the lifetime is that social network size shrinks, but *close* ties remain. This pattern of change is thought to reflect the shift in prioritization of social and emotional goals as people get older, as proposed by the socioemotional selectivity theory [9]. These perspectives suggest that in later life, individuals may be optimizing their networks by focusing more of their social interactions on close ties that are emotionally satisfying.

The quality and quantity of close relationships are robustly associated with individuals’ mental health and well-being [10,11]. One way in which close relationships may benefit psychological well-being is through social support, which increases positive affect, a sense of belonging and self-worth, and buffers the effects of stress [12,13]. Positive relationship quality and interactions with spouse, family, and friends promote mental health and life satisfaction [14–17]. In contrast, living alone is associated with a higher mortality rate in older adults [18]. Furthermore, current research shows that living with a partner prevented a decrease in social connectedness at the early stage of the COVID-19 pandemic [19].

The pandemic’s effect on older adults is multifaceted. Cross-sectional findings indicate that older adults reported better psychological well-being than younger adults [20,21]. This finding is not surprising, given older adults, on average, enjoy better emotional well-being than younger adults [22,23]. However, longitudinal studies show that older adults experienced more depressive and anxiety symptoms and more loneliness during the pandemic when compared to pre-pandemic times [20,24].

### Within-person associations between social interactions and affect

Contemporary research has examined social interactions and affective well-being in day-to-day life with intensive longitudinal assessments, such as daily diaries and ecological momentary assessment (EMA) [25]. Compared to traditional long-term recall surveys, a momentary approach to data collection reduces memory bias and increases ecological validity [25,26]. This body of research provides information about how often individuals are interacting with various social partners or not interacting with anyone. Overall, older adults spend most of their time *not* interacting with other people [27,28].

Perhaps more importantly, the use of repeated momentary assessments allows for the examination of *within-person* associations between social interactions and affect. Despite robust prior evidence using recall surveys showing the benefits of close social relationships for health and well-being, it is unclear whether engaging in meaningful social interactions actually improves immediate well-being; alternatively, it may be the case that healthier and happier individuals are connecting more with others (a between-person association). Prior research has examined subjective well-being during days or moments of social interactions with specific social partners and contrasted it with days or moments not interacting with these social partners regardless of one’s average level of social interaction (a within-person association). Evidence indicates that individuals experience higher positive affect (PA) and lower negative affect (NA) during days and moments when they were interacting with others (see [29] for a review). For instance, adolescents report higher PA on days with higher level of peer support [30]. Individuals feel happier, less sad, more interested, and more socially connected during moments when they are interacting with others than when they are not [10,31]. In a sample of oldest-old adults, moments being with family were associated with higher PA and moments being with friends were associated with both higher PA and lower NA, although within- and between-person effects were not distinguished in the study [27]. Overall, individuals have better well-being during days or moments with social interactions. Although it is often assumed that momentary social interactions bring about higher PA and lower NA, it is also possible that individuals are more likely to engage in social interactions when their PA is higher and their NA is lower.

Some studies have examined momentary solitude and affect. Evidence shows that moments being alone are associated with lower PA, but are not associated with NA in oldest-old adults [27]. Another study showed that momentary solitude was associated with higher levels of low-arousal PA and NA, and lower level of high arousal PA; this suggests that momentary solitude can be both beneficial and detrimental to affective well-being [32]. Nevertheless, research shows that solitude is common among middle-aged and older adults and, for majority of the solitude moments, individuals show desire for solitude [28]. This evidence is consistent with prior conceptualization that solitude is conceptually distinct from loneliness [33].

### The present study

As the United States transitioned from the pre-pandemic period into the pandemic, we expected that there would be significant changes in individuals’ day-to-day life. This study examined how older adults’ amount of momentary solitude, interaction with various social partners (i.e., spouse or significant other, family, friends), and affect (i.e., PA and NA) changed across the early stages of the COVID-19 pandemic, from early March (pre-pandemic) to July 2020 (4 months into the pandemic). We expected NA to increase and PA to decrease after COVID-19 was declared a pandemic. We also expected individuals to have more moments in solitude or interacting with their partner in the same household but fewer interactions with family and friends due to fear of infection. We also examined whether momentary social interactions with various social partners were associated with affect and whether the *magnitude* of associations (i.e., affective benefits associated with social interactions) changed across these early stages of the pandemic. We expected momentary solitude to be associated with lower PA and higher NA, and momentary social interactions with partner, family, and friends to be associated with higher PA and lower NA. We had competing hypotheses regarding whether the magnitude of associations might have changed across time. It is possible that after the declaration of the pandemic, individuals might have valued their time with close ones (i.e., partner, family, friends) more, leading to stronger associations between social interactions and affect. However, it is also possible that these interactions became less enjoyable because of a sudden increase in time spent with their partners as well as fear of virus transmission when with family and friends; thus, this may have led to weakened associations between close relationships and affect. The study used a measurement-burst design [34,35] of EMA to examine these research questions. Participants were assessed at four time points (waves)—early March, late March, May, and July of 2020—across the early stages of the pandemic. During each wave, individuals were prompted six times a day for seven days to report on their social interactions and affect, along with other measures not mentioned here.

## Materials and methods

### Participants and procedure

Participants were from the Understanding America Study (UAS), an ongoing nationally representative Internet panel of respondents aged 18 and older across the U.S. (see https://uasdata.usc.edu/index.php for more information). Panelists aged 50 or older were invited to complete monthly event surveys in the UAS. Those who completed one or more event surveys were invited in cohorts to participate in the burst project, including both EMA and end-of-day surveys. This study focuses on only the EMA assessments. At the end of the invitation email for the burst project, there was a link to a screening survey. Invited panelists were eligible to participate if they: 1) own a smartphone with Android or iOS operating system, and 2) have a monthly voice and data plan associated with their smartphone. Eligible panelists were presented with the study description and were asked to indicate whether they were interested in participating in the study. Panelists who did not respond to the initial invitation email were sent additional reminders. The non-responders remained in the pool of panelists when the study team was ready to invite the next cohort of participants. This study followed two cohorts of participants.

Participants who consented to participate in the study were instructed to download and install the study application. Participants were randomly prompted six times a day for seven days to fill out a short survey. After each prompt, participants had eight minutes to respond to the survey. If they had not responded to the prompt, there was a reminder sent after the initial prompt. Each momentary survey took about two minutes to complete.

Participants received $3 for completing the screening survey and $1 for completing each momentary survey and could receive up to $42 for completing all momentary surveys in a week. Participants were separately compensated for other parts of the study (e.g., end-of-day surveys) not relevant to the current analyses. After the first and second cohorts of participants filled out their surveys in March, they were invited to fill out the same momentary surveys again in May and July. Only participants who completed surveys in either early March or late March were invited to complete the May and July waves.

Each wave of EMA data consists of six measurements a day for seven days (a maximum of 42 prompts for each participant). The first cohort of participants (Cohort A) completed their first wave of EMA in early March (March 2 to March 8) before COVID-19 was declared as a pandemic by the World Health Organization on March 11, 2020, and before any stay-at-home order was issued in the U.S. The second cohort of participants (Cohort B) completed their first wave of EMA in late March (March 23 to March 29), which was after COVID-19 was declared as a pandemic and about when the stay-at-home order was issued for some of the states in the U.S. [36]. Both cohorts completed another wave in May (May 4 to May 10) and another wave in July (July 8 to July 14). The study design is shown in Table 1. The study was approved by the Institutional Review Board at the University of Southern California (UP-14-00148, UP-14-00148-AM087).

**Table 1:** Study design and sample size information.

	Wave 1	Wave 2	Wave 3	Wave 4
Date	March 2–8, 2020	March 23–29, 2020	May 4–10, 2020	July 8–14, 2020
N participants: Cohort A	98	N/A	89	71
N participants: Cohort B	N/A	86	79	64
Total N participants	98	86	168	135
Total N observations	3085	2861	5282	3977

The sample consisted of individuals who provided one or more completed (i.e., with an end timestamp recorded) EMA observations at any wave. Two participants were excluded from analysis due to missing data on a demographic variable used in the analyses. Our final sample consisted of 188 individuals (*M*_*age*_ = 62.05, *SD* = 8.42, range = 50–88). Among the participants, 60% were female, 82% identified as White only, 66% were living with their spouse or partner, 43% had a bachelor’s degree, and 53% were currently working. Participants identified their marital status as married (61%), separated or divorced (21%), widowed (8%), and never married (10%). The median household income was $60,000–$99,999. Participants were comparable to 2019 census data for a similar age group regarding median household income, marital status, and the percentage of White individuals, but participants were more likely to be female (compared to 53% female in census) and more likely to hold a Bachelor’s degree (compared to 32% in census) [37,38]. The two cohorts did not significantly differ in their demographic characteristics (see results section). Out of 23,688 possible EMA observations, participants provided 15,205 completed observations, with each person on average providing 80.88 observations across waves.

### Measures

#### Momentary affect

At each prompt, participants were asked how much they were feeling the following affect before the prompt: *frustrated*, *dejected/blue/downhearted*, *stressed*, *angry*, *lonely*, *happy*, *cheerful*, and *relaxed*. Participants rated each affective state on a visual analogue scale (VAS) from 0–*not at all* to 100–*extremely*. Scores from items with positive adjectives were averaged to indicate the level of PA and scores from items with negative adjectives were averaged to indicate the level of NA at every momentary prompt. Reliability of PA and NA were assessed using the between-person reliability estimate, R_1F_, and the reliability estimate of within-person change, R_c_ [39]. Both scales exhibited adequate between-person and within-person reliability (R_1F_ ranged from .91 to .93 for PA and ranged from .81 to .94 for NA across waves; R_c_ ranged from .72 to .81 for PA and was .76 for NA across waves).

#### Momentary social interactions

At each prompt, participants were asked to indicate who they were interacting with before the prompt from the list of social partners: no one, spouse or significant other (hereafter we referred to as partner), family, friends, public, supervisor, co-worker, and other. Participants were instructed to select all that applied. As the present study focused on close relationships, only data pertaining to *no one*, *partner*, *family*, and *friends* were analyzed. Each item was coded as 0–*no* and 1–*yes* for each social partner. If a participant interacted with more than one social partner at a particular moment, those social interactions were coded as 1 at that specific moment. No one refers to solitude, with 1–*yes* indicating individuals not having interactions with *any* of the seven social partners (i.e., including both close and distant relationships) at a particular moment.

#### Demographics

The demographic information was collected in a separate questionnaire (i.e., My Household Survey) from the larger UAS. The survey regularly collected demographic information from the UAS panelists and the information that was collected most proximal to participants’ first EMA wave was used. It included participants’ age, gender, race, cohabitation, education, work status, and income. Gender was coded as 0–*female* and 1–*male*. Race was coded as 0–*non-White or mixed race* and 1–*White only*. Living with spouse or partner was coded as 0–*not living with spouse or partner* and 1–*living with spouse or partner*. Education was coded as 0–*no bachelor’s degree* and 1–*with a bachelor’s degree or above*. Work status was coded as 0–*not currently working* and 1–*currently working*. Annual household income was coded as 0–*less than $30*,*000*, 1–*$30*,*000 - $59*,*999*, 2–*$60*,*000 - $99*,*999*, and 3–*$100*,*000 or more*.

#### Analysis plan

The study was not specially designed to investigate the COVID-19 pandemic as it was part of a previously scheduled data collection effort. Therefore, some aspects of the design were not ideal. Only Cohort A participated in the first wave in early March (before the declaration of the pandemic and before stay-at-home order was issued for any state in the U.S.), and only Cohort B participated in the second wave in late March (when stay-at-home orders were in effect for some of the states). However, both cohorts participated in May and in July. Because we were going to use data from both cohorts to examine how momentary social interactions and affect changed from early March through July, we determined if there were sample differences between the two cohorts. First, we examined whether or not the two cohorts differed in their demographic characteristics. We conducted t-tests and chi-square tests to examine whether the two cohorts differed in age, gender, race, living with spouse or partner, education, work status, and income. Second, we examined whether the two cohorts had comparable affective well-being and social interactions in May and July using t-tests. Third, in order to further minimize possible cohort differences, we controlled for demographic characteristics in all of the main analyses. All analyses were conducted in *R* version 4.1.1. Multilevel analyses were conducted with the *lme4* package version 1.1–27 [40] and the *lmerTest* package version 3.1–3 [41] with restricted maximum likelihood estimation. Follow-up contrasts were conducted using the *emmeans* package version 1.7.1–1 [42].

For main analyses, we first examined whether momentary social interactions (i.e., with no one, partner, family, and friends) changed from March to July. Using three-level generalized multilevel models, we tested whether “wave” as a four-level variable predicted the log odds of interacting with 1) no one, 2) partner, 3) family, and 4) friends while controlling for age, gender, race, living with spouse or partner, education, working status, and income. In these models, the observations were nested within waves within individuals, and random intercepts were allowed. A sample model equation (control variables not shown) is:

$$\begin{array}{l} P\;({Social\;Interaction}_{twi}=1)=\frac{\exp\left(\eta_{twi}\right)}{1+\exp\left(\eta_{twi}\right)}\\ \mathrm{Level}\;1\;\mathrm{Moments}:\;\eta_{twi}=\beta_{0wi}\\ \mathrm{Level}\;2\;\mathrm{Waves}:\;\beta_{0wi}=\delta_{00i}+\delta_{01i}{Wave}_{wi}+u_{0wi}\\ \mathrm{Level}\;3\;\mathrm{Persons}:\;\delta_{00i}=\gamma_{000}+v_{00i}\\ \;\delta_{01i}=\gamma_{010} \end{array}$$

In this model, *t* indicates moment at level 1, *w* indicates wave at level 2, and *i* indicates person at level 3. To examine the overall effect of wave on the log odds of various social interactions, likelihood ratio test was used to compare models with and without “wave” as a predictor. A significant chi-square statistic indicated that the log odds of that specific social interaction significantly differed across waves. For models with a significant chi-square statistic, follow-up contrasts were used to identify significant differences in the log odds of social interactions between each pair of waves; results were presented in predicted probabilities to facilitate interpretation. The default Tukey method adjustment for multiple comparisons was used.

In the second set of analyses, we examined whether momentary affect (PA and NA) changed from March to July 2020 in two separate models. Using three-level multilevel models, we examined whether wave predicted affect while controlling for the same demographic variables as in previous models. Again, the observations were nested within waves within individuals, and we allowed for random intercepts. The model equation for PA is:

$$\begin{array}{l} \mathrm{Level}\;1\;\mathrm{Moments}:\;{PA}_{twi}=\beta_{0wi}+e_{twi}\\ \mathrm{Level}\;2\;\mathrm{Waves}:\;\beta_{0wi}=\delta_{00i}+\delta_{01i}{Wave}_{wi}+u_{0wi}\\ \mathrm{Level}\;3\;\mathrm{Persons}:\;\delta_{00i}=\gamma_{000}+v_{00i}\\ \;\delta_{01i}=\gamma_{010} \end{array}$$

The same model was used for NA. To examine the overall effect of wave on each affect, the F-test with Satterthwaite’s method was used. A significant F-test indicated that the means of the respective affect significantly differed across waves. For models with a significant F-test, follow-up contrasts were used to identify the pairwise waves in which there were significant differences in affect.

In the third set of analyses, we examined whether momentary social interactions were associated with affective well-being within wave within person. Using multilevel models, we examined whether momentary social interaction predicted affect (i.e., PA and NA), while controlling for individuals’ average level of the respective social interaction within a wave, wave, and demographic characteristics. As the binary social interaction predictor at level 1 was uncentered, by including the within-wave person mean (i.e., proportion) of the respective social interaction as a predictor, the estimated effect of momentary social interactions on affect (β_1wi_ in the following equation) indicates the within-wave *within-person association* between social interaction and affect [43–45]. The within-wave person mean social interaction at level 2 was grand-mean centered. Observations were nested within waves within individuals. In addition, we allowed for random intercepts and random slopes of momentary social interaction. A sample model equation for PA is:

$$\begin{array}{l} \mathrm{Level}\;1\;\mathrm{Moments}:\;{PA}_{twi}=\beta_{0wi}+\beta_{1wi}{Social\;Interaction}_{twi}+e_{twi}\\ \mathrm{Level}\;2\;\mathrm{Waves}:\;\beta_{0wi}=\delta_{00i}+\delta_{01i}{Wave}_{wi}+\delta_{02i}{Social\;Interaction\;Prop}_{wi}+u_{0wi}\\ \;\beta_{1wi}=\delta_{10i}+u_{1wi}\\ \mathrm{Level}\;3\;\mathrm{Persons}:\;\delta_{00i}=\gamma_{000}+v_{00i}\\ \;\delta_{01i}=\gamma_{010}\\ \;\delta_{02i}=\gamma_{020}\\ \;\delta_{10i}=\gamma_{100}+v_{10i} \end{array}$$

The variance in momentary affect explained by each momentary social interaction (or solitude) was calculated as the percentage reduction in the residual variance of the model from a model without the predictor β_1wi_ Social Interaction_twi_.

In the fourth set of analyses, we examined if the magnitude of associations between momentary social interactions and affective well-being changed across waves from March to July. Using multilevel models, we examined how momentary social interaction, wave, and their interaction term predicted affect (i.e., PA and NA), while controlling for individuals’ average level of the respective social interaction within a wave (see explanation for the prior set of models) and demographic characteristics. Again, observations were nested within waves within individuals. In addition, we allowed for random intercepts and random slopes of momentary social interaction. A sample model equation for PA is:

$$\begin{array}{l} \mathrm{Level}\;1\;\mathrm{Moments}:\;{PA}_{twi}=\beta_{0wi}+\beta_{1wi}{Social\;Interaction}_{twi}+e_{twi}\\ \mathrm{Level}\;2\;\mathrm{Waves}:\;\beta_{0wi}=\delta_{00i}+\delta_{01i}{Wave}_{wi}+\delta_{02i}{Social\;Interaction\;Prop}_{wi}+u_{0wi}\\ \;\beta_{1wi}=\delta_{10i}+\delta_{11i}{Wave}_{wi}+u_{1wi}\\ \mathrm{Level}\;3\;\mathrm{Person}:\;\delta_{00i}=\gamma_{000}+v_{00i}\\ \;\delta_{01i}=\gamma_{010}\\ \;\delta_{02i}=\gamma_{020}\\ \;\delta_{10i}=\gamma_{100}+v_{10i}\\ \;\delta_{11i}=\gamma_{110} \end{array}$$

To examine the interaction between momentary social interaction and the overall effect of wave in predicting affect, the F-test with Satterthwaite’s method was used. A significant F-test indicated that the association between the respective momentary social interaction and affect significantly differed across waves. For models with a significant F-test, follow-up contrasts were used to identify pairwise waves in which significant differences in association occurred. The Benjamini-Hochberg adjustment for multiple comparisons was applied [46].

For all four sets of analyses, the same set of demographic characteristics were used as control variables; age was centered at 50 and scaled by 1/10 (i.e., in the unit of decades) to facilitate model convergence. For models examining interactions with partner, we only included individuals who lived with their spouse or partner in the analyses. As a result, living with spouse or partner was not included as a control variable in those analyses.

As the NA composite contains the item “lonely,” there is a potential risk of conflating solitude and NA. Therefore, we conducted sensitivity analyses for all the models involving NA and solitude by excluding the “lonely” item.

## Results

Before conducting the main analyses, we first examined whether the two cohorts differed on major demographic characteristics. Results from t-tests and chi-square tests indicated that the two cohorts did not differ significantly on any of the demographic characteristics (i.e., age, gender, race, living with spouse or partner, education, working status, and income). Furthermore, results from t-tests showed that the two cohorts did not differ significantly in individuals’ mean levels of affect (PA and NA) and proportions of social interactions (i.e., with no one, partner, family, and friends) in May and July, except that individuals in Cohort B had significantly more family interactions than those in Cohort A in July (Mean_A_ = 0.13; Mean_B_ = 0.21, *t*(122.06) = -2.20, *p* = .030). Together, the evidence suggests that the two cohorts were likely to be random samples of the same population. In order to further minimize possible cohort differences, we controlled for demographic characteristics in all of our main analyses.

### Did momentary social interactions change across waves?

**Fig 1:**
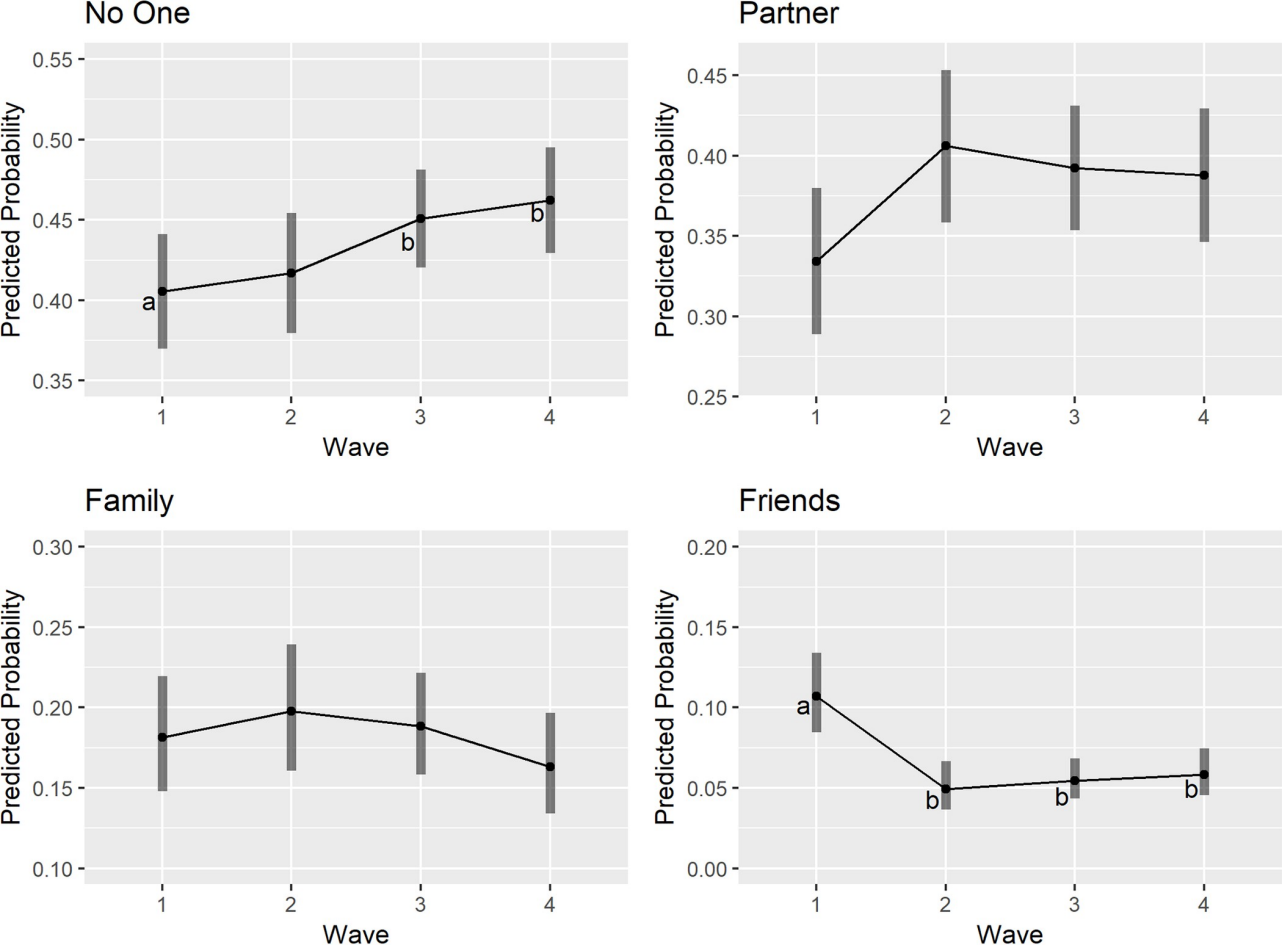
The predicted probabilities of various momentary social interactions across waves. *Note*. For models with a significant overall effect of wave, different letters indicate that the predicted probabilities in those waves were significantly different from each other.

For the main analyses, we first examined whether momentary social interactions (i.e., with no one, partner, family, and friends) changed across the four waves (see Table 2). After controlling for demographic characteristics, the overall effect of wave on solitude was significant (*X*^2^ (3) = 17.64, *p* < .001). Follow-up contrasts showed that the predicted probability of solitude was significantly higher in Wave 3 (45.1%) and Wave 4 (46.2%) compared to Wave 1 (40.5%; Wave 3/Wave 1: *OR* = 1.93, *SE* = 0.17, *p* = .034; Wave 4/Wave 1: *OR* = 2.05, *SE* = 0.20, *p* = .009). For partner interactions and family interactions, results indicated that they did not differ significantly across waves (partner: *X*^2^(3) = 7.54, *p* = .056; family: *X*^2^(3) = 5.47, *p* = .141). Finally, interaction with friends differed significantly across waves (*X*^2^ (3) = 25.50, *p* < .001). Results from follow-up contrasts indicated that the predicted probabilities of interacting with friends at Wave 2 (4.9%; *OR* = 0.72, *SE* = 0.14, *p* < .001), Wave 3 (5.5%, *OR* = 0.80, *SE* = 0.12, *p* < .001), and Wave 4 (5.8%; *OR* = 0.86, *SE* = 0.14, *p* < .001) were significantly lower than that at Wave 1 (10.7%). The predicted probabilities of momentary social interactions across waves are shown in Fig 1.

**Table 2:** Multilevel models with wave and demographic variables predicting the log odds of social interactions.

	No One		Partner		Family		Friends	
N observations	15,201		10,295		15,201		15,201	
N participants	188		124		188		188	
	Estimate	SE	Estimate	SE	Estimate	SE	Estimate	SE
**Fixed effects**								
Intercept	0.62*	0.29	-1.29**	0.50	-1.53***	0.41	-2.68***	0.37
Wave 2	0.12	0.12	0.37*	0.16	0.09	0.15	-0.79***	0.20
Wave 3	0.26**	0.09	0.32**	0.12	0.02	0.12	-0.72***	0.15
Wave 4	0.36***	0.09	0.27*	0.13	-0.17	0.13	-0.57***	0.16
*Control variables*								
*Age*	-0.26**	0.10	0.56***	0.15	-0.34*	0.14	0.07	0.13
*Gender (male)*	-0.01	0.16	0.20	0.21	-0.79***	0.22	-0.03	0.20
*Race (white)*	0.15	0.20	-0.38	0.33	0.01	0.28	0.60*	0.25
*Living with spouse or partner*	-1.09***	0.18	--	--	0.00	0.25	-0.99***	0.23
*College*	0.08	0.17	-0.39	0.25	-0.26	0.24	0.03	0.21
*Working*	-0.05	0.17	-0.30	0.24	-0.30	0.25	-0.60**	0.22
*Income 1 ($30*,*000–$59*,*999)*	-0.31	0.22	0.51	0.37	0.14	0.31	0.54	0.27
*Income 2 ($60*,*000–$99*,*999)*	-0.39	0.26	0.32	0.38	0.72*	0.37	0.20	0.33
*Income 3 (> = $100*,*000)*	-0.21	0.25	0.21	0.36	0.71*	0.36	0.71*	0.33
**Random effects**								
Intercept Var: wave:person	0.23		0.29		0.35		0.52	
Intercept Var: person	0.79		0.98		1.56		0.90	
AIC	18289.09		11800.48		11820.23		7085.72	
BIC	18403.53		11901.83		11934.67		7200.16	
*R*^*2*^ conditional	0.29		0.34		0.40		0.36	
*R*^*2*^ marginal	0.07		0.09		0.05		0.08	

Note.

* *p* < .05

** *p* < .01

*** *p* < .001. Wald tests were conducted only on fixed effects. Var = variance.

### Did momentary affect change across waves?

**Fig 2:**
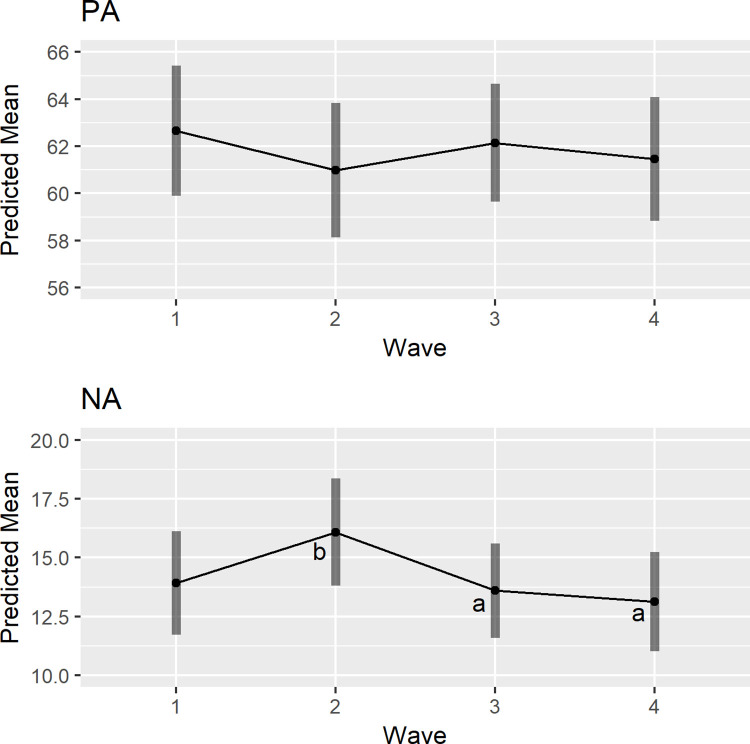
The predicted means of momentary PA and NA across waves. *Note*. For models with a significant overall effect of wave, different letters indicate that the predicted means in those waves were significantly different from each other.

Next, we examined whether momentary affect (PA and NA) changed across the four waves from early March to July, 2020 using three-level multilevel models. Results are shown in Table 3. After controlling for demographic characteristics, the overall effect of wave on PA was not significant (*F*(3, 305.16) = 1.49, *p* = .217), indicating that PA was not significantly different across waves. The same model was conducted for NA. Results showed that the overall effect of wave on NA was significant (*F*(3, 309.76) = 4.50, *p* = .004) and follow-up contrasts indicated that NA means at Wave 3 (mean = 13.60; z = -2.48, SE = 0.83, *p* = .015) and Wave 4 (mean = 13.14; z = -2.94, SE = 0.88, *p* = .004) were significantly lower than NA at Wave 2 (mean = 16.08). The predicted means of momentary affect across waves are shown in Fig 2.

**Table 3:** Multilevel models with wave and demographic variables predicting PA and NA.

	PA		NA	
N observations	15,193		15,192	
N participants	188		188	
	Estimate	SE	Estimate	SE
**Fixed effects**				
Intercept	55.55***	4.63	18.95***	3.70
Wave 2	-2.26	1.33	2.53*	1.05
Wave 3	-0.81	1.00	-0.19	0.79
Wave 4	-1.84	1.05	-0.41	0.83
*Control variables*				
*Age*	4.70**	1.66	-1.85	1.32
*Gender (male)*	5.11*	2.56	0.46	2.05
*Race (white)*	-4.83	3.28	2.23	2.62
*Living with spouse or partner*	3.61	2.96	-4.53	2.37
*College*	-2.46	2.75	-0.97	2.20
*Working*	3.07	2.86	-0.11	2.29
*Income 1 ($30*,*000–$59*,*999)*	0.03	3.58	-1.20	2.86
*Income 2 ($60*,*000–$99*,*999)*	1.07	4.27	-3.30	3.42
*Income 3 (> = $100*,*000)*	1.49	4.20	-1.59	3.36
**Random effects**				
Intercept Var: wave:person	45.72		28.07	
Intercept Var: person	242.96		155.91	
Residual Var	101.70		70.21	
AIC	115083.75		109406.89	
BIC	115205.81		109528.95	
*R*^*2*^ conditional	0.76		0.73	
*R*^*2*^ marginal	0.06		0.03	

*Note*.

* *p* < .05

** *p* < .01

*** *p* < .001. Wald tests were conducted only on fixed effects. Var = variance.

### Were momentary social interactions associated with affect?

Next, we examined whether momentary social interactions were associated with affect. Results from multilevel models predicting PA and NA are shown in Tables 4 and 5, respectively. After controlling for wave, the within-wave person mean of the respective social interaction, and demographic characteristics, momentary solitude predicted lower PA (B = -1.46, SE = 0.30, *p* < .001) and momentary interactions with partner, family, and friends all predicted higher PA (partner: B = 1.91, SE = 0.38, *p* < .001; family: B = 3.19, SE = 0.41, *p* < .001; friends: B = 3.43, SE = 0.51, *p* < .001). For NA, after controlling for wave, the within-wave person mean of the respective social interaction, and demographic characteristics, momentary solitude was not significantly associated with NA (B = 0.16, SE = 0.25, *p* = .514). However, momentary interactions with partner, family, and friends all predicted lower NA (partner: B = -1.28, SE = 0.32, *p* < .001; family: B = -1.50, SE = 0.32, *p* < .001; friends: B = -1.98, SE = 0.39, *p* < .001). The variance in PA explained by each momentary social interaction was: no one (3.24%), partner (3.18%), family (4.71%), and friends (2.08%). The variance in NA explained by each momentary social interaction was: no one (3.63%), partner (3.39%), family (3.66%), and friends (2.27%).

**Table 4:** Multilevel models with various social interactions and wave predicting PA.

	No One		Partner		Family		Friends	
N observations	15,189		10,284		15,189		15,189	
N participants	188		124		188		188	
	Estimate	SE	Estimate	SE	Estimate	SE	Estimate	SE
**Fixed effects**								
Intercept	53.81***	4.55	55.62***	6.88	53.97***	4.62	54.73***	4.58
Momentary social interaction	-1.46***	0.30	1.91***	0.38	3.19***	0.41	3.43***	0.51
Wave 2	-1.82	1.33	-1.97	1.53	-2.23	1.33	-1.82	1.36
Wave 3	-0.54	1.00	0.57	1.14	-0.82	0.99	-0.33	1.03
Wave 4	-1.30	1.06	-0.30	1.20	-1.83	1.05	-1.47	1.07
Social interaction proportion	-2.32	2.75	1.28	3.14	-1.86	3.34	6.09	5.06
*Control Variables*								
*Age*	4.63**	1.62	5.04*	2.14	4.78**	1.65	4.27**	1.64
*Gender (male)*	5.53*	2.50	5.10	2.96	5.22*	2.56	4.93	2.53
*Race (white)*	-1.96	3.20	-3.73	4.72	-3.96	3.26	-4.66	3.25
*Living with spouse or partner*	3.21	2.95	--	--	3.80	2.95	4.58	2.95
*College*	-3.76	2.68	-5.34	3.44	-2.64	2.74	-2.53	2.72
*Working*	3.75	2.79	6.29	3.42	3.70	2.85	3.18	2.84
*Income 1 ($30*,*000–$59*,*999)*	0.70	3.50	0.55	5.11	0.03	3.56	0.20	3.54
*Income 2 ($60*,*000–$99*,*999)*	0.81	4.17	0.12	5.34	0.63	4.26	1.51	4.23
*Income 3 (> = $100*,*000)*	1.26	4.09	1.85	5.08	1.13	4.18	1.24	4.16
**Random effects**								
Intercept Var: wave:person	52.28		40.59		46.31		46.15	
Slope Var: wave:person	8.60		9.27		30.80		14.65	
Intercept Var: person	227.57		240.78		245.21		243.15	
Slope Var: person	6.21		5.72		2.89		9.44	
Residual Var	98.40		102.56		96.91		99.58	
AIC	114845.57		78105.11		114676.19		114892.46	
BIC	115013.40		78257.11		114844.01		115060.29	
*R*^*2*^ conditional	0.76		0.75		0.77		0.76	
*R*^*2*^ marginal	0.07		0.08		0.06		0.06	

*Note*.

* *p* < .05

** *p* < .01

*** *p* < .001. Wald tests were conducted only on fixed effects. Var = variance.

**Table 5:** Multilevel models with various social interactions and wave predicting NA.

	No One		Partner		Family		Friends^+^	
N observations	15,188		10,283		15,188		15,188	
N participants	188		124		188		188	
	Estimate	SE	Estimate	SE	Estimate	SE	Estimate	SE
**Fixed effects**								
Intercept	21.16***	3.73	11.77*	5.20	19.59***	3.70	18.37***	3.63
Momentary social interaction	0.16	0.25	-1.28***	0.32	-1.50***	0.32	-1.98***	0.39
Wave 2	2.71**	1.03	2.31	1.24	2.46*	1.05	3.00**	1.05
Wave 3	0.22	0.78	-0.65	0.93	-0.46	0.78	0.11	0.79
Wave 4	0.12	0.82	-0.64	0.98	-0.47	0.83	0.05	0.82
Social interaction proportion	-7.59***	2.16	-0.30	2.52	6.04*	2.61	-1.18	3.79
*Control Variables*								
*Age*	-2.22	1.33	-2.67	1.63	-1.67	1.32	-1.37	1.30
*Gender (male)*	0.20	2.05	0.86	2.24	0.45	2.05	0.90	2.01
*Race (white)*	1.82	2.63	4.75	3.58	1.76	2.62	2.24	2.58
*Living with spouse or partner*	-6.33**	2.42	--	--	-4.31	2.37	-5.43*	2.34
*College*	-0.60	2.20	-1.27	2.60	-0.88	2.20	-0.74	2.16
*Working*	-0.11	2.29	-0.75	2.59	-0.27	2.29	0.10	2.25
*Income 1 ($30*,*000–$59*,*999)*	-2.08	2.87	0.14	3.86	-1.35	2.86	-1.34	2.81
*Income 2 ($60*,*000–$99*,*999)*	-4.26	3.43	0.12	4.03	-3.56	3.42	-3.45	3.36
*Income 3 (> = $100*,*000)*	-2.17	3.36	1.50	3.83	-1.85	3.35	-1.26	3.30
**Random effects**								
Intercept Var: wave:person	28.68		29.50		31.02		30.15	
Slope Var: wave:person	10.22		8.09		18.25		18.28	
Intercept Var: person	155.40		122.25		156.21		157.40	
Slope Var: person	2.39		3.39		1.25		0.65	
Residual Var	67.66		67.69		67.64		68.62	
AIC	109171.90		73838.94		109126.92		109197.60	
BIC	109339.72		73990.95		109294.74		109365.42	
*R*^*2*^ conditional	0.75		0.70		0.74		0.74	
*R*^*2*^ marginal	0.04		0.04		0.04		0.04	

*Note*.

* *p* < .05

** *p* < .01

*** *p* < .001. Wald tests were conducted only on fixed effects. Var = variance.

^+^The solution of this model resulted in a singular covariance matrix, but the solution provided by the *nlme* package in R [47] was admissible and results were practically the same. Therefore, original results were presented.

### Did the magnitude of associations between momentary social interactions and affect change across waves?

Finally, we examined whether the magnitude of associations between momentary social interactions and affect differed across waves using multilevel models. Results from multilevel models predicting PA and NA are shown in Tables 6 and 7, respectively. After controlling for demographic characteristics and the within-wave person mean of respective social interaction, momentary solitude significantly interacted with the overall effect of wave to predict PA (*F*(3, 308.44) = 4.39, *p* = .005). Results from follow-up contrasts showed that the magnitude of negative association between solitude and PA at Waves 1 and 3 was significantly larger than that at Wave 2 (Wave 1 –Wave 2: *z* = -2.11, *SE* = 0.86, *p* = .042; Wave 3 –Wave 2: *z* = -2.00, *SE* = 0.74, *p* = .041). Momentary family interaction also significantly interacted with the overall effect of wave to predict PA (*F*(3, 265.94) = 3.94, *p* = .009). Results from follow-up contrasts showed that the magnitude of associations between momentary family interaction and PA at Waves 1 and 3 was significantly larger than that at Wave 2 (Wave 1 –Wave 2: *z* = 3.38, *SE* = 1.35, *p* = .037; Wave 3 –Wave 2: *z* = 3.35, *SE* = 1.15, *p* = .022). Further, momentary friend interaction significantly interacted with the overall effect of wave to predict PA (*F*(3, 163.11) = 2.68, *p* = .049). Results from follow-up contrasts showed that the magnitude of associations between momentary friend interaction and PA at Wave 1 was significantly larger than that at Waves 2, 3, and 4 (Wave 1 –Wave 2: *z* = 4.49, *SE* = 1.65, *p* = .018; Wave 1 –Wave 3: *z* = 3.32, *SE* = 1.27, *p* = .018; Wave 1 –Wave 4: *z* = 3.46, *SE* = 1.30, *p* = .018). However, momentary partner interaction did not significantly interact with wave to predict PA (*F*(3, 194.84) = 1.59, *p* = .194)

**Table 6:** Multilevel models with various social interactions, wave, and social interactions by wave predicting PA.

	No One		Partner		Family		Friends	
N observations	15,189		10,284		15,189		15,189	
N participants	188		124		188		188	
	Estimate	SE	Estimate	SE	Estimate	SE	Estimate	SE
**Fixed effects**								
Intercept	54.58***	4.55	55.23***	6.85	53.81***	4.62	54.49***	4.57
Momentary social interaction	-2.62***	0.56	3.09***	0.73	4.00***	0.86	5.38***	0.86
Wave 2	-3.29*	1.40	-1.10	1.60	-1.58	1.35	-1.53	1.37
Wave 3	-0.90	1.08	0.97	1.19	-0.81	1.01	-0.11	1.04
Wave 4	-2.37*	1.14	0.32	1.25	-1.71	1.06	-1.21	1.07
Social Interaction:wave 2	2.51**	0.80	-2.10*	1.04	-3.53**	1.24	-3.11*	1.41
Social Interaction:wave 3	0.69	0.66	-1.05	0.87	0.02	1.07	-2.41*	1.11
Social Interaction:wave 4	1.79*	0.70	-1.60	0.91	-0.67	1.16	-2.91*	1.20
Social interaction proportion	-2.47	2.75	1.39	3.14	-1.73	3.34	6.14	5.05
*Control Variables*								
*Age*	4.55**	1.61	5.01*	2.13	4.76**	1.65	4.17*	1.63
*Gender (male)*	5.56*	2.49	5.10	2.95	5.22*	2.56	4.90	2.53
*Race (white)*	-1.95	3.19	-3.78	4.70	-3.88	3.26	-4.52	3.24
*Living with spouse or partner*	3.25	2.94	--	--	3.82	2.95	4.61	2.94
*College*	-3.88	2.67	-5.42	3.42	-2.63	2.74	-2.56	2.71
*Working*	3.65	2.78	6.36	3.40	3.67	2.85	3.14	2.83
*Income 1 ($30*,*000–$59*,*999)*	0.67	3.49	0.55	5.09	0.01	3.56	0.28	3.53
*Income 2 ($60*,*000–$99*,*999)*	1.02	4.15	0.28	5.32	0.63	4.26	1.63	4.22
*Income 3 (> = $100*,*000)*	1.26	4.08	1.80	5.06	1.08	4.18	1.29	4.15
**Random effects**								
Intercept Var: wave:person	51.99		40.52		46.33		46.14	
Slope Var: wave:person	7.99		9.17		30.47		13.94	
Intercept Var: person	226.62		241.94		245.42		243.29	
Slope Var: person	6.37		5.83		1.98		9.39	
Residual Var	98.39		102.55		96.90		99.58	
AIC	114835.99		78102.30		114665.02		114884.35	
BIC	115026.70		78276.02		114855.73		115075.06	
*R*^*2*^ conditional	0.76		0.75		0.77		0.76	
*R*^*2*^ marginal	0.07		0.08		0.06		0.06	

*Note*.

* *p* < .05

** *p* < .01

*** *p* < .001. Wald tests were conducted only on fixed effects. Var = variance.

**Table 7:** Multilevel models with various social interactions, wave, and social interactions by wave predicting NA.

	No One		Partner		Family		Friends^+^	
N observations	15,188		10,283		15,188		15,188	
N participants	188		124		188		188	
	Estimate	SE	Estimate	SE	Estimate	SE	Estimate	SE
**Fixed effects**								
Intercept	21.10***	3.73	11.96*	5.21	19.69***	3.70	18.87***	3.62
Momentary social interaction	0.35	0.51	-1.64**	0.62	-1.50*	0.68	-3.79***	0.73
Wave 2	3.02**	1.07	2.05	1.32	1.92	1.09	2.18*	1.09
Wave 3	0.13	0.82	-0.74	1.00	-0.27	0.82	-0.53	0.83
Wave 4	0.31	0.87	-0.98	1.05	-0.39	0.86	-0.65	0.86
Social Interaction:wave 2	-0.72	0.73	0.55	0.90	1.63	0.98	3.05*	1.20
Social Interaction:wave 3	0.18	0.62	0.22	0.74	-0.67	0.85	2.18*	0.98
Social Interaction:wave 4	-0.44	0.65	0.70	0.78	-0.30	0.91	2.54*	1.05
Social interaction proportion	-7.55***	2.16	-0.34	2.52	6.01*	2.61	-1.35	3.76
*Control Variables*								
*Age*	-2.21	1.33	-2.67	1.63	-1.65	1.32	-1.26	1.29
*Gender (male)*	0.18	2.05	0.86	2.24	0.40	2.05	0.95	2.00
*Race (white)*	1.80	2.63	4.73	3.58	1.64	2.62	2.13	2.57
*Living with spouse or partner*	-6.34**	2.42	--	--	-4.29	2.36	-5.47*	2.32
*College*	-0.59	2.20	-1.28	2.60	-0.92	2.19	-0.68	2.15
*Working*	-0.10	2.29	-0.77	2.59	-0.29	2.28	0.13	2.23
*Income 1 ($30*,*000–$59*,*999)*	-2.09	2.87	0.13	3.87	-1.36	2.85	-1.39	2.79
*Income 2 ($60*,*000–$99*,*999)*	-4.29	3.43	0.13	4.04	-3.51	3.41	-3.47	3.35
*Income 3 (> = $100*,*000)*	-2.18	3.36	1.50	3.84	-1.83	3.35	-1.28	3.28
**Random effects**								
Intercept Var: wave:person	28.70		29.51		30.96		30.06	
Slope Var: wave:person	10.30		8.28		18.06		18.02	
Intercept Var: person	155.19		122.52		156.51		157.64	
Slope Var: person	2.39		3.40		1.25		0.85	
Residual Var	67.66		67.68		67.63		68.61	
AIC	109173.28		73840.76		109121.55		109189.28	
BIC	109363.99		74014.48		109312.26		109379.99	
*R*^*2*^ conditional	0.75		0.70		0.74		0.74	
*R*^*2*^ marginal	0.04		0.04		0.03		0.04	

*Note*.

* *p* < .05

** *p* < .01

*** *p* < .001. Wald tests were conducted only on fixed effects. Var = variance.

^+^The solution of this model resulted in a singular covariance matrix, but the solution provided by the *nlme* package in R [47] was admissible and results were practically the same. Therefore, original results were presented.

**Fig 3:**
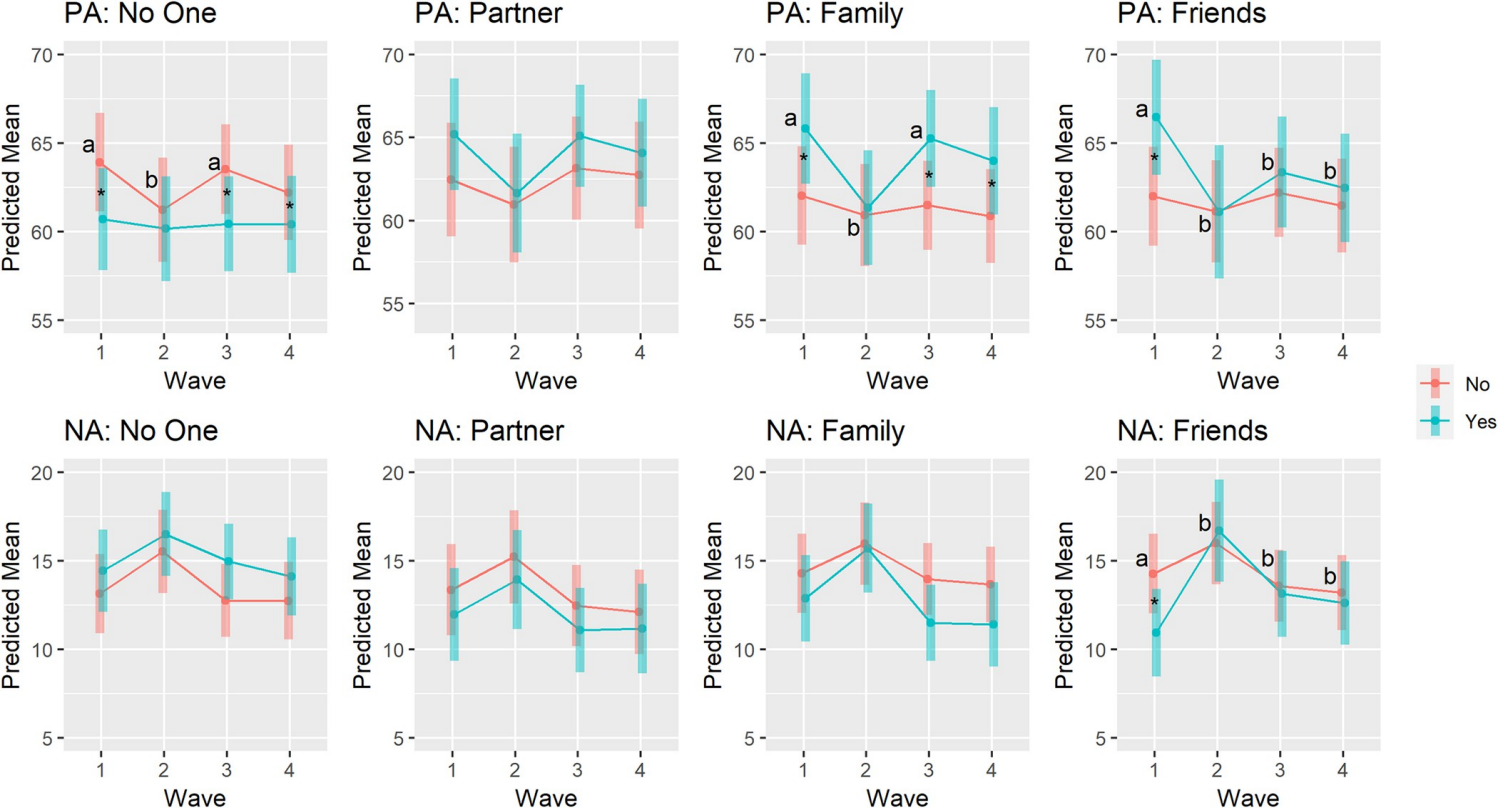
PA and NA predicted by momentary social interactions across waves. *Note*. For models with a significant “momentary social interaction × wave” interaction, * indicates that the difference in affect between with and without social interaction was significant at a specific wave and different letters indicate that the differences in affect between with and without social interaction in those waves were significantly different from each other.

For NA, momentary solitude, partner interaction, family interaction did not significantly interact with the overall effect of wave to predict NA (solitude: *F*(3, 361.11) = 0.82, *p* = .481; partner: *F*(3, 204.53) = 0.33, *p* = .804; family: *F*(3, 296.78) = 2.44, *p* = .064). However, momentary friend interaction significantly interacted with wave to predict NA (*F*(3, 336.52) = 3.04, *p* = .029). Results from follow-up contrasts showed that the magnitude of negative associations between momentary friend interaction and NA at Wave 1 was significantly larger than that at Waves 2, 3, and 4 (Wave 1 –Wave 2: *z* = -4.03, *SE* = 1.37, *p* = .020; Wave 1 –Wave 3: *z* = -2.87, *SE* = 1.09, *p* = .026; Wave 1 –Wave 4: *z* = -2.72, *SE* = 1.12, *p* = .031). The affect predicted by momentary social interactions across waves is shown in Fig 3.

We conducted sensitivity analyses for all models involving NA and solitude by excluding the “lonely” item from the NA scale to verify that our findings were not due to the conflation of solitude and loneliness. The results were practically the same.

## Discussion

This study examined how momentary social interactions and affect changed from the period before the declaration of the COVID-19 as a pandemic through four months into the pandemic in a sample of older adults. There were several key findings. First, with whom people spent their time changed. Older adults spent increasingly more time in solitude at two months and four months into the pandemic compared to the pre-pandemic period despite the fact that most, if not all, stay-at-home orders were lifted. Similarly, older adults spent significantly less time interacting with friends after the declaration of the pandemic, and again, the level of interactions did not rebound to pre-pandemic level. The time individuals spent with partners and family did not change significantly across time. Second, levels of NA changed. The level of NA was the highest right after the pandemic was declared but returned to pre-pandemic level thereafter. In contrast, PA did not change significantly across the study period. Third, momentary social interactions with one’s partner, family, and friends were consistently associated with higher level of PA and lower level of NA. Momentary solitude was associated with lower PA, but was not related to NA. Finally, the affective benefits associated with momentary social interactions and the detriments associated with a lack of social interactions changed in magnitude across the study period. Specifically, right after the declaration of the pandemic, solitude was not associated with lower PA and interactions with family were not associated with higher PA as they were before the pandemic and two months after its declaration. Also, higher levels of PA and lower levels of NA associated with friend interactions before the pandemic were diminished following its declaration.

A consistent finding of the present study was that social interactions were closely related to affective well-being at the within-person level: moments interacting with close social ties (i.e., partner, family, and friends) were consistently associated with higher level of PA and lower level of NA compared to moments when individuals were not interacting with them. This is consistent with existing theories that close social interaction is a fundamental human need and that older adults’ close social relationships are generally emotionally satisfying [4,9]. Our findings also echo the empirical *within-person* evidence that social interactions are central to well-being [29], which also held true during the first few months of the COVID-19 pandemic [48,49]. It is also important to note that moments not interacting with anyone were associated with lower PA, but not higher NA. This suggests that solitary moments are less positive, which could be due to a lack of positive social stimuli, but those moments were not necessarily more negative. This finding is consistent with prior evidence that solitary moments in oldest-old adults are not experienced negatively [27].

Although we expected older adults to be solitary more often after the onset of the pandemic, we were surprised to find that this was not the case during the heightened phase of the pandemic (late March). However, it was the case at two and four months into the pandemic when most, if not all, stay-at-home orders were lifted. Relatedly, older adults interacted significantly less often with their friends since the pandemic declaration and those interactions did not rebound even when the stay-at-home orders were lifted. It is possible that older adults were reluctant to interact with others outside of the family, even when social restrictions were gradually loosened, because they felt more vulnerable to severe symptoms of infection. Another explanation may be that as individuals adapt to social distancing rules and spend more time alone, they discover routines and hobbies they prefer to perform alone. Future research should examine if this is the case.

It was also surprising that NA only increased during the heightened phase of the pandemic and then returned to pre-pandemic level, given the expected increase in social distancing fatigue [50]. A possible explanation is that despite the significant changes in everyday life introduced by the pandemic, individuals emotionally adapted to it over time. The finding is consistent with the idea that individuals have certain set points of well-being and despite changes in the environment, most people adapt to these circumstances over time [51]. However, it is important to point out that our findings represent the overall average of the population being studied. There were likely subgroups of individuals (e.g., individuals with family members who had COVID-19) who might fare worse as the pandemic progressed.

It is also notable that our findings showed a somewhat different picture of well-being compared with other studies of mental health symptoms before and during the pandemic. For instance, other research has shown that adults were more than three times likely to be screening positive for anxiety and depressive disorders in April to May, 2020 as compared to the prior year [52] and older adults experienced increased loneliness and depressive symptoms during April to May, 2020 as compared to months before the pandemic [53]. There are a few possible explanations for the discrepancies. First, it could be that most people have adapted psychologically to the circumstances of the pandemic, but subgroups of individuals experienced more mental health symptoms over time. Relatedly, it is possible that individuals who were experiencing more severe distress might not have participated in our study. Second, it is possible that individuals in early March were already experiencing higher than usual NA and our study failed to capture the shift in NA early enough. Finally, momentary affect and mental health symptoms may reflect different facets of well-being and the pandemic impacts them differently.

Another notable finding was that the magnitude of association between certain social interactions and affect in older adults changed significantly across the early stages of the pandemic, and the onset of the pandemic appeared to be a critical point. During presumably the most stressful period (late March, 2020), the usual association between momentary solitude and lower PA was diminished, suggesting that being solitary during this time was not necessarily less positive. It is possible that moments of solitude entail less worry about getting infected. Furthermore, higher level of PA usually associated with momentary family interactions was diminished during the same period, suggesting family interactions during the most stressful time of the pandemic were not particularly rewarding. It is possible that the contexts in which family interactions took place might have changed due to COVID-19 (e.g., from family parties to physically distanced walks or online chats) or that individuals might have worried about infecting their families or being infected during family gatherings, given the health consequences of infection were more severe in older adults. Nevertheless, the affective benefits appeared to have been restored by two months into the pandemic.

Finally, the affective benefits (higher PA and lower NA) associated with interactions with friends before the pandemic was diminished following the onset of the pandemic, and unlike family interaction, the benefits did not rebound despite the gradual loosening of social restrictions. Overall, the findings suggest older adults were interacting significantly less often with friends following the onset of the pandemic, and, when they did, their interactions appeared less rewarding compared to pre-pandemic times. It is possible that older adults might have experienced more infection-related worry when interacting with friends—not only are friends likely from a different household, but participants might also have less knowledge about their friends’ social lives than those of their partner or family.

There are several limitations in the present study. First, we were not able to distinguish in-person from virtual social interactions (e.g., Facetime, Zoom). It is plausible that these two types of social interactions were differentially associated with momentary affect, which is a topic for future study. Second, participants in early March and late March were different samples. Although our findings suggest that they were likely to be sampled from the same population and we controlled for demographic characteristics in all of the analyses, it remains a limitation of the study. Third, as stated previously, the earliest data we have on individuals were in early March. This was before COVID-19 was declared as a pandemic and before any state has initiated stay-at-home order, yet there is a possibility that individuals’ well-being was already affected by COVID-19 as its threat was looming. Thus, the “pre-pandemic” time might not be the optimal reference point for comparing social interactions and affect to those during the pandemic. Fourth, we did not have information about whether individuals were working in-person or from home. Individuals who worked from home were likely to have more time alone or interacting with people in the same household (e.g., partner). Fifth, there were potential selection biases in study participation: a) individuals working from home might have had more time to participate in the current EMA study; b) individuals who were White and more highly educated might have been more likely to participate as shown in prior research [54]; and c) individuals with lower income might have been less likely to own a smartphone (a requirement for the present study). Nevertheless, the current sample is comparable to the U.S. census data regarding household income and the percentage of White individuals. Finally, other important factors such as infection rates and financial stability could have impacted individuals’ affect level, but these factors were not examined in the study.

In conclusion, this study has shown that momentary social interactions with close social ties are correlated with older adults’ affective well-being, regardless of their average amount of social interactions with them. Older adults, on average appeared to be emotionally resilient across the early stages of the pandemic. The magnitude of associations between affect and specific social interactions (or solitude) changed across the study period in which the pandemic declaration appeared to be a critical time point. Additionally, older adults spent increasingly more time not interacting with others and continued to spend less time interacting with friends despite stay-at-home orders were gradually being lifted throughout the country. It is unclear when the pattern of social interactions will return to “normal” or if the changes will be lasting that there will be a new “normal” for social interactions, even after the pandemic has subsided.
